# One if By Hand, Two if Orally: PICCing the Best Treatment Option

**DOI:** 10.1093/ofid/ofad663

**Published:** 2024-01-12

**Authors:** Monica V Mahoney, Christina F Yen

**Affiliations:** Beth Israel Deaconess Medical Center, Pharmacy Department, Boston, Massachusetts, USA; Maine Medical Center, Division of Infectious Diseases, Portland, Maine, USA

**Keywords:** OPAT, oral antibiotics, transitions of care, Bacteremia

Commercial health insurance claims databases can give us a wealth of information, hence their increasing attractiveness as a data source for clinical, pharmacoepidemiologic, and other forms of research. While individual institutions can perform retrospective analyses on their own patient populations, oftentimes this results in underpowered studies that cannot answer the clinical question at hand. However, given access to claims databases, the potential to investigate outcomes in thousands of patients over vast geographic areas exists. But, in exchange for an increased number of eligible patients, new limitations emerge, with several worth serious consideration. First, these databases mostly cover outpatient claims. Therefore, relevant inpatient details may be censored, unless coded and available in the database. Particularly with subjectively defined outcomes, the accuracy and validity of some databases may be called into question. Second, these databases prioritize data relevant to insurance companies, not clinicians. Details like a patient's home milieu or overall status, which are variables health care professionals regularly factor into the calculus of clinical decision-making, are not captured.

Such is the case in this month's OFID article by Hamad and colleagues, which uses the Merative Marketscan Commercial Database to compare outcomes in patients with methicillin-susceptible *Staphylococcus aureus* (MSSA) bloodstream infections (BSIs) discharged on outpatient parenteral antimicrobial therapy (OPAT) on ceftriaxone or the traditional agents of nafcillin, oxacillin, or cefazolin (“comparator”) [1]. The outcomes of interest were 90-day hospital readmission with the same infection category and 90-day all-cause readmission. The authors conclude that there was no statistically significant difference between readmission for either end point in patients receiving ceftriaxone or comparator during OPAT for MSSA BSI.

There are many strengths of the study. This is the largest retrospective study to date, with 460 patients receiving ceftriaxone and 1435 receiving a comparator antibiotic, a scale that would unlikely be readily or promptly achievable through retrospective review of a single institution or health care system's clinical databases. Infection types were well balanced and represented infections commonly treated with OPAT, including skin and soft tissue infections (40.0%), osteomyelitis (17.7%), septic arthritis (13.3%), and prosthetic joint infection (8.6%). Imbalances were seen in patients with epidural abscesses and endocarditis receiving comparator antibiotics more frequently and patients with pneumonia receiving ceftriaxone more frequently. Patients had comparable inpatient stays before receiving OPAT (7 days ceftriaxone vs 8 days comparator; *P* < .001) and identical OPAT durations (median, 15 days each). The outcomes evaluated, death and readmission, are objective end points, and therefore less likely to be influenced by subjective coding. Based on this study, one could relatively confidently state that patients with MSSA BSI who are ready for discharge on OPAT would have similar outcomes if they were discharged on ceftriaxone, nafcillin, oxacillin, or cefazolin.

Why is this important? For patients being discharged home, the burden of medication administration and line care mainly lies with them or a family member. Patients may receive care from a visiting nurse once per week, but otherwise need to be trained to infuse medications and maintain the line. For medications with multiple doses per day, this can encompass several hours per day. Most home infusion medications require refrigeration for stability. Therefore, the patient must remove the medication anywhere from 15–60 minutes before infusion to allow the medication to come to room temperature [2]. Coupled with the amount of time required to properly sanitize, infuse, and flush the lines, this can become cumbersome. Meanwhile, patients and their support systems must schedule other necessary medical care such as follow-up appointments around these hours of logistics. It is therefore unsurprising that there are studies showing that as the number of infusions per day increases, the rate of adherence decreases. While 1–2 infusion per day have adherence rates of 76%, 3 or more infusions per day result in 26% adherence [3]. Oxacillin and nafcillin are dosed 6 times per day, cefazolin is dosed 3 times per day, and ceftriaxone is only dosed once per day (assuming normal renal function for all). The attractiveness of a daily-dose antimicrobial lies not only in ease of administration, but also in its potential to reduce morbidity and mortality from dangerous MSSA BSI–associated complications by increasing adherence to optimal therapy as well.

However, a major limitation of the current study is the inability to discern how a hospitalized patient is deemed ready for discharge. We already established that when a patient is ready for discharge on OPAT, any of the investigated regimens may be suitable. But what aspects, clinical or otherwise, determine safety and readiness? We may presume from our own clinical experience that this includes duration of antimicrobial therapy, achievement of source control, blood culture clearance, and hemodynamic stability. What about fine motor coordination to administer therapy and manage one's line, family engagement, language comprehension, access to refrigeration, or geographic proximity to health care should complications with OPAT arise? Another limitation of the study is the nonrandomized nature, as this is a retrospective review. There were noted differences in terms of treatment selection for endocarditis, epidural abscesses, and pneumonia. There may be additional confounders unable to be evaluated, such as the selection of patients who were discharged to skilled nursing facilities, rehabilitation centers, or long-term care facilities. As this study only reviewed patients who were discharged *home* on the antibiotics of their choice, did more acutely ill, more complex, sicker patients get discharged to locations other than home, and were they therefore unable to be included in the study?

Unfortunately, much of the aforementioned information of interest is not available in the health insurance database. On average, patients were hospitalized for 7 (ceftriaxone) and 8 (comparator) days before discharge. While we do not know how many days of inpatient antibiotic therapy they received, it can be assumed that the duration may be similar if not slightly lower than the duration of inpatient stay. All the patients had bloodstream infections, meaning that staphylococci were detected in blood cultures. These are rarely deemed contaminates and therefore are usually promptly treated. Unfortunately, follow-up information such as rates and duration until blood culture clearance or source control was unavailable and was mentioned as a limitation by the authors. While hemodynamic stability information and other nuances are missing, it can be assumed that they were met, as patients were discharged. However, the absence of definitive data here invites room for further interrogation into how clinicians determine discharge and OPAT readiness.

Lastly, if patients met clinician criteria for hospital discharge, another emerging question would be—why do they need intravenous antibiotics? Could they not have been discharged on oral antibiotics? There is growing evidence that a switch to oral therapy, even for infections such as staphylococcal BSIs, is effective. The POET trial demonstrated that this is feasible for gram-positive endocarditis [4]. Numerous retrospective studies are solidifying the role of oral linezolid [5–7]. Intravenous therapy, even a regimen consisting of a once-daily administration, still comes with placement of an intravenous line and the risks of line-associated events [8, 9]. By utilizing the oral route, these risks are bypassed. Oral therapy may not be an option for all patients—intravenous options may be the only susceptible medications, swallowing or gastrointestinal complications may impact administration and absorption of oral dosage forms, and drug interactions or adverse effects may preclude use. But for those who qualify, oral routes can be much less burdensome than intravenous while allowing patients to potentially attend school or work and carry on other daily activities without compromising the quality of their care [10, 11].

The study by Hamad and colleagues is a welcome addition to the growing data on use of ceftriaxone in the treatment of MSSA infections via OPAT. Ceftriaxone has many advantages as an OPAT medication compared with oxacillin, nafcillin, and cefazolin. But there are still lingering questions that OPAT clinicians would like answered. When is a patient ready for OPAT discharge? What considerations, both clinical and otherwise, are most important to ascertain safety and readiness for OPAT discharge? What is the ideal intravenous OPAT option for MSSA BSI? What is the optimal ceftriaxone dose? What is the ideal duration of therapy? And what is the ideal route for outpatient MSSA BSI treatment? These last questions cannot be answered by large retrospective studies. A prospective, randomized, active controlled study would need to be conducted. The question is—who is willing to conduct this study to shed light on our way forward in this condition?
